# Preliminary Quantitative Evaluation of the Optimal Colour System for the Assessment of Peripheral Circulation from Applied Pressure Using Machine Learning

**DOI:** 10.3390/s25144441

**Published:** 2025-07-16

**Authors:** Masanobu Tsurumoto, Takunori Shimazaki, Jaakko Hyry, Yoshifumi Kawakubo, Takeshi Yokoyama, Daisuke Anzai

**Affiliations:** 1 Department of Clinical Engineering, Faculty of Health and Welfare, Tokushima Bunri University, Kagawa 760-8542, Japan; tsurumoto@kgw.bunri-u.ac.jp; 2 Department of Clinical Engineering, Faculty of Health Care, Jikei University of Health Care Sciences, Osaka 532-0003, Japan; 3 Graduate School of Informatics, Osaka Metropolitan University, Osaka 599-8531, Japan; 4 Department of Dental Anesthesiology, Faculty of Dental Science, Kyushu University, Fukuoka 812-8582, Japan

**Keywords:** peripheral circulation, bed sores, pressure ulcers, skin tone, colour space components, machine learning

## Abstract

Peripheral circulatory failure refers to a condition in which the blood flow through superficial capillaries is markedly reduced or completely occluded. In clinical practice, nurses strictly adhere to regular repositioning protocols to prevent peripheral circulatory failure, during which the skin condition is evaluated visually. In this study, skin colour changes resulting from pressure application were continuously captured using a camera, and supervised machine learning was employed to classify the data into two categories: before and after pressure. The evaluation of practical colour space components revealed that the h component of the JCh colour space demonstrated the highest discriminative performance (Area Under the Curve (AUC) = 0.88), followed by the a* component of the CIELAB colour space (AUC = 0.84) and the H component of the HSV colour space (AUC = 0.83). These findings demonstrate that it is feasible to quantitatively evaluate skin colour changes associated with pressure, suggesting that this approach can serve as a valuable indicator for dimensionality reduction in feature extraction for machine learning and is potentially an effective method for preventing pressure-induced skin injuries.

## 1. Introduction

Peripheral circulatory failure refers to a condition caused by a significant decrease or blockage of blood flow in capillaries in the superficial layer. If it continues for a long time, the tissue under the skin will necrose and develop into a bedsore. The main causes are the patient’s own weight and pressure from medical equipment, and tissue damage during reperfusion and also known to be involved in the onset of bedsores. To prevent bedsores, it is important to disperse pressure from compression, so frequent position changes are recommended. However, in the medical field, when nurses do these position changes regularly, they visually judge skin colour and judge the bedsores using the DESIGN-R classification, and quantitative standards for evaluating the condition before bedsores are not yet well established. Establishing this system would reduce the number of times patients need to change positions and the incidence of bedsores, as this would contribute to improving the workload of nurses and the quality of medical care. Colour spaces that numerically evaluate the hue, saturation, and brightness perceived by humans are widely used as methods for evaluating colour, and various colour spaces have been proposed for different purposes [1,2].

Pinto et al. attempted clinical evaluation using peripheral circulation, which is non-invasive and highly reproducible, instead of the invasive systemic hemodynamic monitoring used in intensive care units, and demonstrated the prognosis prediction of critically ill patients and the effectiveness of shock resuscitation using capillary refill time (CRT), skin temperature, and mottle score as indicators [3]. Xia et al. compared and verified the evaluation of CRT, which is one of the microcirculatory monitoring methods recommended by the International Sepsis Guidelines, the American Academy of Pediatrics, the World Health Organization (WHO), and the American Heart Association (AHA), by compressing the surface of the skin with a certain pressure and measuring the time it takes for the skin colour to return to its original state after blood is expelled; semi-automatic measurement, which analyses changes in skin colour using a digital camera and visual feedback technology to quantify CRT; and fully automatic measurement, which uses pressure sensors and photoplethysmography (PPG) technology to electronically measure the compression and recovery process, demonstrating the effectiveness of the fully automatic measurement method [4]. Li et al. combined hemodynamic parameters such as blood pressure and cardiac output, microcirculatory markers such as skin colour and temperature, tissue oxygen saturation, and laser Doppler blood flow measurement, with metabolic indices such as near-infrared spectroscopy (NIRS), central venous oxygen saturation (ScvO_2_), and lactate levels to comprehensively evaluate macro- and microcirculatory and metabolic states, accurately evaluating tissue perfusion and predicting patient prognosis [5].

Rozhkova et al. applied the asymmetric colour matching (ACM) method to a smartphone to simply and effectively evaluate colour perception in the peripheral visual field, and showed the relationship with the eccentric angle and stimulus luminance expressed in the HSV colour space [6]. Zhang et al. used imaging photoplethysmography (IPPG) technology to compare the effects of colour spaces and colour formats such as RGB, HSV, and YCbCr, and showed that a combination of a specific colour space and colour format can reduce interference from environmental light and facial movement and improve measurement accuracy [7]. Kaur et al. compared the performance of YCbCr and CIELAB colour spaces in skin colour segmentation and demonstrated that the CIELAB colour space has device-independent and stable discrimination ability against illumination changes, and demonstrated that it is possible to distinguish skin more accurately than the YCbCr colour space [8]. Oghaz et al. proposed a new three-dimensional hybrid colour space ’SKN’ to improve the accuracy of skin and face detection, and searched for the optimal skin colour representation using genetic algorithm (GA) and principal component analysis (PCA). In pixel-by-pixel skin detection, the SKN colour space using Random Forest achieved higher accuracy (F-score 0.95, TPR 0.95, FPR 0.048) than conventional colour spaces, and was shown to be the best classifier [9]. Abbas et al. proposed a new pattern classification method to distinguish benign nevi from malignant melanoma in dermatological diagnosis. By utilising the features of the uniform colour space of benign skin and the non-uniform texture of malignant skin, and by using a multi-label learning algorithm with adaptive boosting, they achieved high classification performance of 89.28% sensitivity (SE), 93.75% specificity (SP), and AUC = 0.98, which was consistent with the diagnosis by dermatologists [10].

While in research on peripheral circulation in clinical evaluation, non-invasive measurement methods using CRT, skin temperature, and mottle scores as indicators have been proposed, and comprehensive evaluation methods integrating hemodynamic parameters, microcirculatory markers, and metabolic indicators have also been considered. Quantification of skin colour using colour spaces, improvement of skin classification accuracy by comparing different colour spaces, and utilisation of colour and texture in dermatological diagnosis are being promoted. However, there has been no research to date that examines the subtle colour changes that occur due to pressure changes using colour spaces, and examines each component of the spaces that would be effective for machine learning.

Furthermore, many of the aforementioned methods suffer from limited temporal resolution due to signal smoothing, manual frame annotation, or reliance on average brightness recovery. For example, Akimbekov et al. demonstrated that video-based semi-automatic CRT measurements are constrained by inter-observer variability and frame-based temporal limitations. Verkruysse et al. also noted that iPPG-based techniques, although suitable for tracking periodic signals, lack responsiveness to brief, transient changes in skin perfusion. These limitations highlight the need for alternative techniques capable of capturing rapid skin tone fluctuations immediately following pressure release [11,12].

In contrast, the method proposed in this study enables frame-level classification without reliance on periodicity or contact-based sensors, and may offer improved robustness under controlled imaging conditions. These characteristics suggest its potential utility in clinical applications that require non-invasive, rapid assessment of peripheral circulatory changes.

In order to non-invasively evaluate changes in peripheral circulation due to pressurisation, the purpose of this study is to establish a proof of concept for quantitatively analysing colour changes on the skin surface and identify optimal colour space components and their features in a controlled setting. Furthermore, by applying these components as features to a machine learning model and evaluating the classification performance, we consider the optimal combination of components that accurately reflect changes in peripheral circulation. This study uses supervised machine learning to classify skin colour changes caused by pressure into two classes, before and after pressure, and identifies effective colour space components.

## 2. Theoretical Background

### 2.1. Colour Spaces

#### 2.1.1. RGB

RGB (Red Green Blue) is a basic colour space for representing colour information in digital images, and is widely used in the fields of digital image processing and computer vision [13]. In this model, each pixel is decomposed into three channels, red, green, and blue, and the brightness value of each channel is defined as a range from 0 to 255. This range is generally represented by 8-bit integer values, which allows the reproduction of a total of about 16.77 million colours [14]. By appropriately combining the values of the three channels, a wide range of colours that can be perceived by humans can be represented. The RGB colour system in OpenCV is usually based on the sRGB (Standard Red Green Blue) colour space, which is an international standard colour space defined by the International Electrotechnical Commission (IEC) and standardised as IEC 61966-2.1 [15]. This colour space is used by many digital devices and image file formats, such as computer displays, digital cameras, and printers. Using sRGB as the standard enables consistent colour reproduction across different devices, achieving accurate colour expression that is not dependent on a specific environment or device. Furthermore, when calculating the average colour of an entire image or a specific area using RGB values, the average RGB value for the entire area can be obtained by calculating the average value for each channel. This calculation is expressed by the following formula.

$${\mathrm{Mean}}_{R}=\frac{\sum_{(i,j)\in P}R\left(i,j\right)}{N}$$

$${\mathrm{Mean}}_{G}=\frac{\sum_{(i,j)\in P}G\left(i,j\right)}{N}$$

$${\mathrm{Mean}}_{B}=\frac{\sum_{(i,j)\in P}B\left(i,j\right)}{N}$$

- *R*: Average of red pixel values within Region of Interest (ROI);
- *G*: Average of green pixel values within ROI;
- *B*: Average of blue pixel values within ROI;
- *i*: Row index of pixel;
- *j*: Column index of pixel;
- *P*: Set of all pixel coordinates contained in the area defined as ROI;
- *N*: Total number of pixels within ROI.

Although it is easy to calculate and is suitable for light-emitting devices such as displays, it has the disadvantage that the appearance is non-uniform, and it is difficult to calculate the colour difference because it does not take into account the characteristics of human vision. To solve this problem, HSV was devised to make it easier to use for design purposes. Furthermore, CIELAB, which represents a perceptually uniform colour space, and JCh, which represents a colour expression that is more faithful to the visual sense, were devised [16].

#### 2.1.2. HSV

**Figure 1:**
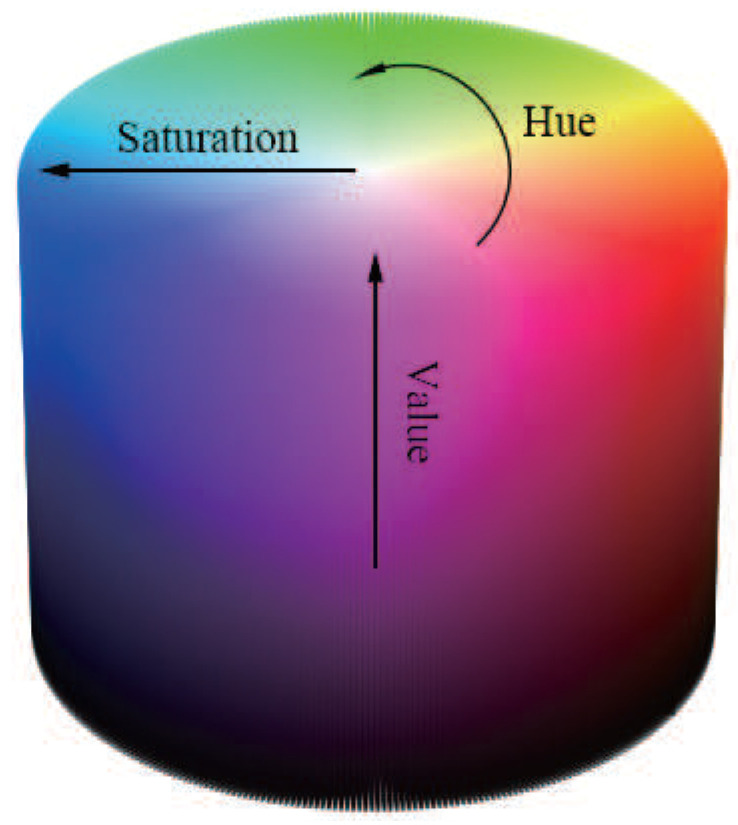
HSV chromaticity diagram.

The HSV (Hue, Saturation, Value) colour space expresses colours from a different perspective than the RGB colour space, and is designed based on human visual colour perception [17,18]. This colour space is composed of three elements: hue, saturation and value as shown in Figure 1, which are defined as follows. Hue represents the colour tone and is defined in the range from 0to 360. For example, 0corresponds to red, 120to green, and 240to blue. This allows the types of colours to be expressed in a circular ring [19]. Saturation represents the vividness of the colour and is defined in the range from 0% to 100%. At 0% it is gray (achromatic), and at 100% it is the most vivid colour. The higher the value, the purer the colour, and the lower the value, the closer to gray it is [20]. Value represents the brightness of a colour and is defined in the range from 0% to 100%. Higher values are brighter and lower values are darker. 0% is completely black and 100% is the brightest state of that hue [18]. The average HSV value is expressed by the following formula:

$${\mathrm{Mean}}_{H}=\frac{\sum_{(i,j)\in P}H\left(i,j\right)}{N}$$

$${\mathrm{Mean}}_{S}=\frac{\sum_{(i,j)\in P}S\left(i,j\right)}{N}$$

$${\mathrm{Mean}}_{V}=\frac{\sum_{(i,j)\in P}V\left(i,j\right)}{N}$$

- *H*: Average of Hue pixel values within ROI;
- *S*: Average of Saturation pixel values within ROI;
- *V*: Average of Value pixel values within ROI;
- *i*: Row index of pixel;
- *j*: Column index of pixel;
- *P*: Set of all pixel coordinates contained in the area defined as ROI;
- *N*: Total number of pixels within ROI.

#### 2.1.3. CIELAB

**Figure 2:**
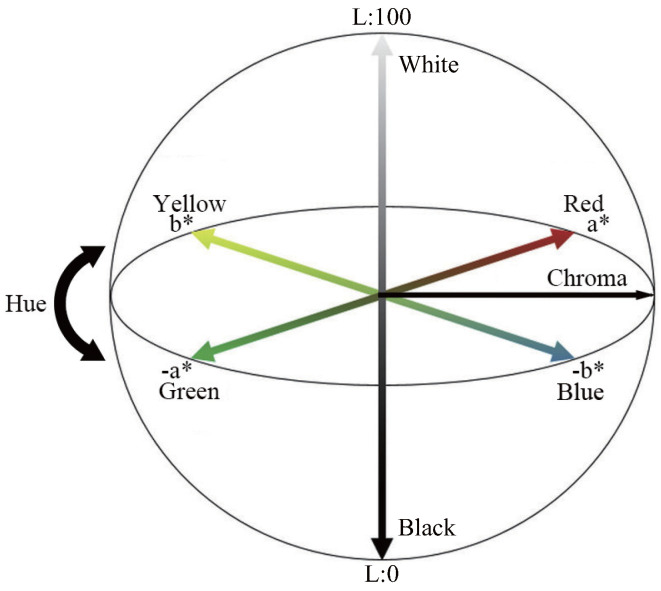
CIELab colour space.

The CIELAB (CIE L*a*b*) colour space was established in 1976 by the International Commission on Illumination (CIE) as a standard model for numerically expressing colours based on human visual characteristics. It is composed of a three-dimensional Cartesian coordinate system of lightness (L∗), red-green components (a∗), and yellow-blue components (b∗). This colour space is device-independent and can accurately describe a wide range of colours, making it widely used in a wide range of fields, including colour management and colour difference calculations. As shown in Figure 2, L∗ represents the brightness of the colour. Its value ranges from 0 to 100, with 0 representing black and 100 representing white. b∗ represents the hue between yellow and blue. Positive values are more yellowish, and negative values are more blueish. These three axes are configured as a Cartesian coordinate system, and each colour is uniquely defined in the colour space [16]. In addition, when a∗ and b∗ are 0, the colour is achromatic (gray). CIE L*a*b* is device-independent, so it can describe all colours that humans perceive, providing consistent colour performance across different displays and printers. It also covers a wider range than RGB and can express almost all colours that humans can perceive. In addition, it is designed as a uniform colour space, so that visual colour differences are reflected as numerical distances. Nonlinear transformation (1/3 power) is applied to lightness and colour components, reproducing responses based on human visual characteristics [21,22]. In OpenCV, CIELAB components are treated as 8-bit integers, so the values are scaled and expressed using the following formula [23]:

$$L*=\frac{L_{\mathrm{scaled}}}{255}\times 100$$

$$a*=a_{\mathrm{scaled}}-128$$

$$b*=b_{\mathrm{scaled}}-128$$

- L*: Range scaled from 0 to 255;
- a*: Range scaled from 0 to 255;
- b*: Range scaled from 0 to 255.

#### 2.1.4. JCh

The JCh colour space is a colour space designed based on the CIE Colour Appearance Model 2002 (CIECAM02), and expresses colour in three elements: lightness (J), chroma (C), and hue (h). This colour space was developed based on the CIE LCh colour space, and has perceptually uniform characteristics [24]. CIECAM02 is a model that aims to reproduce the appearance of colours based on human visual characteristics, and is particularly useful in evaluating perceptual colour differences and colour gamut mapping [25]. By utilising the characteristics of this model, the JCh colour space provides more uniform perceptual colour differences, and is expected to be applied in the fields of colour management and image processing [26].

Lightness J is an index that indicates the brightness of a colour and is defined in the range from 0 to 100. Within this range, 0 represents complete black and 100 represents the brightest white. Lightness is based on the intensity of light as perceived by humans, and is a numerical representation of the visual sense of brightness [27]. Saturation C is an index of the vividness and intensity of a colour, with higher values representing more vivid colours and lower values representing gray. Saturation reflects the purity and vividness of a colour as perceived by humans, with low saturation approaching neutral colours (gray) and high saturation being perceived as purer colours [28]. Hue h is an element that indicates colour tone and is defined as an angle from 0° to 360°. For example, red is 0°, green is 120°, and blue is 240°; hue is expressed in a circular ring [26]. This angular representation reflects the continuity of colour perception by humans, and allows intuitive and visual understanding of the relationship between different hues. In addition, by adopting a polar coordinate system, saturation C is expressed as a radius from the origin, and hue h is expressed as an angle on a circle. This format improves the ability to capture subtle changes in hue, achieving highly accurate classification performance [25].

The JCh colour space is designed to accurately reflect the colour difference perceived by humans as a numerical distance, making it possible to quantitatively evaluate the differences between different colours [29]. This perceptual uniformity is based on the basic theory of CIECAM02, and faithfully reproduces the characteristics of human vision [25]. In addition, this colour space is device-independent, allowing consistent colour management across different devices [30]. Thanks to these features, the JCh colour space combines intuitive visual expression with high accuracy, and is widely used as the basis for advanced colour management systems that take into account human visual characteristics [27].

The JCh value is expressed using the following formula:

$$J=100\cdot {\left(\frac{A}{A_{w}}\right)}^{c\cdot z}$$

$$C=\sqrt{a^{2}+b^{2}}$$

$$H=\arctan\left(\frac{b}{a}\right)$$

- *A*: Achromatic response—achromatic component of the observed object;
- $A_{w}$: White point of the Achromatic response—achromatic component of the white reference;
- *c*: Scaling coefficient considering the surrounding conditions;
- *z*: Degree of adaptation—usually defined as $z=1.48+\sqrt{n}$ (where $n=Y_{b}/Y_{w}$, the relative luminance ratio between the background and white).

### 2.2. Fundamental Evaluation for Non-Invasive Detection of Early Circulatory Changes Using Colour Space Analysis

**Figure 3:**
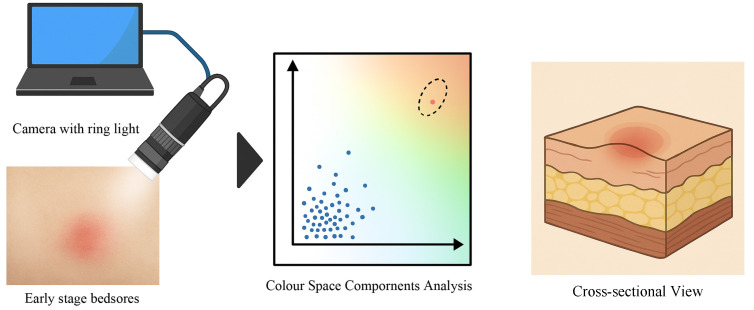
Pressure ulcer analysis model using colour space components.

This study is a fundamental evaluation to verify whether minute circulatory changes in the skin caused by external pressure can be quantitatively captured using camera-based imaging and colour space analysis. By establishing the ability to non-invasively monitor these early circulatory changes, the goal is to detect early-stage pressure ulcers that are difficult to detect by conventional visual inspection, as illustrated in Figure 3.

## 3. Experimental Environment

### 3.1. Protocols

**Figure 4:**
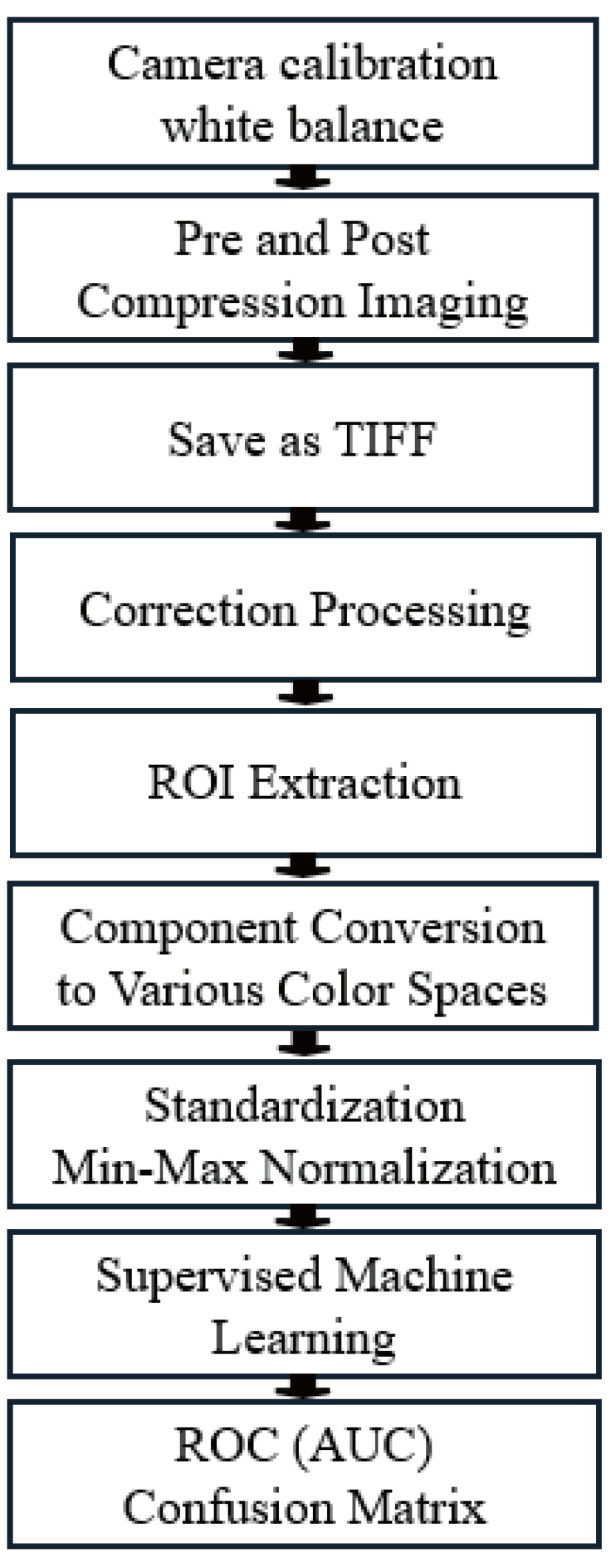
Experimental protocol.

**Figure 5:**
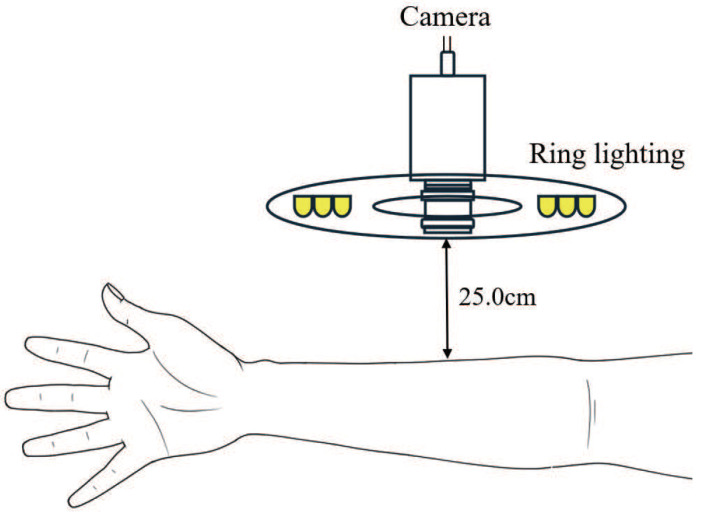
Camera and ring light diagram.

Figure 4 shows the processing procedure from data collection to evaluation. Twenty healthy adults without hypertension or circulatory disorders were photographed while resting in a supine position. The experiment was conducted in an indoor environment without external light, using a ring light (brightness = 5300 k) that is less likely to cast shadows, and a medical colour camera with a shooting speed of 15 fps (frames per second) fixed on a tripod. The colour camera was white balanced using a Macbeth chart (X-Rite Color Checker Classic^®^) in the Basler pylon viewer 8.0.1 tool, and the shooting distance was set to the closest position from the upper arm where no halation occurs, which was 25.0 cm in this study, as illustrated in Figure 5.

**Figure 6:**
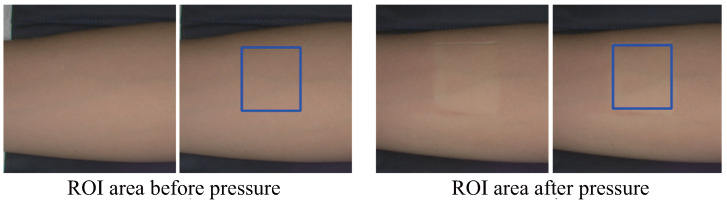
ROI area, indicated by the blue rectangle, before and after applying pressure.

A blood pressure measurement cuff was wrapped around the forearm, and a 37 × 47 × 20 mm plate was placed on the palm side of the forearm under the cuff, and this position was set as the region of interest. Imaging was then perfomed with the following steps: We performed a ten second imaging before applying pressure, followed by 30 s of pressure application with the compression set to 80 mmHg, and lastly, after pressure imaging for 60 s, we had a total imaging time of 100 s, as shown in Figure 6. To ensure safety, 80 mmHg was set as the maximum value based on the minimum systolic blood pressure (90 mmHg), while also taking into consideration the subject’s physical and mental stress and making sure to not completely block the blood flow.

As preprocessing, the area from the wrist to the upper arm was trimmed to 1000 × 500 pixels for all files saved in TIFF format, and slight tilt deviations caused by body movement were corrected using the wrist and elbow as reference points. After that, the area compressed by the plate was designated as the ROI, and a rectangle of 130 × 140 pixels was cut out. The brightness values of red (R), green (G), and blue (B) were obtained in the RGB colour space, and the hue, saturation, and value were calculated in the HSV colour space. In addition, lightness (L*), red-green component (a*), and blue-yellow component (b*) were calculated in the CIELAB colour space, and lightness (J), saturation (C), and hue angle (h) were calculated in the JCh colour space based on the CIECAM02 colour appearance model. These 12 components were used as objective variables and standardised. The abbreviations are shown in Table 1.

To define the classification objective variable, a single representative frame was extracted from each subject. Class 0 was defined using a frame captured under stable baseline conditions before cuff inflation, representing the resting state of peripheral circulation, while Class 1 was defined using a frame captured immediately after cuff release, corresponding to the early reperfusion phase. Frame selection was performed according to a standardized protocol across all subjects, ensuring consistency and physiological comparability. This approach allowed for the construction of a balanced dataset focused on the representative skin colour transition induced by pressure application and release. Finally, supervised machine learning (ensemble bagging) was used to perform 5-fold cross-validation, and the top components in the AUC obtained were evaluated using ROC curves and standard classification metrics, including AUC, precision, recall, and F1 score. Statistical significance of the results was also assessed using appropriate hypothesis testing.

To construct the classification model, we employed an ensemble bagging algorithm using decision trees as base learners. Based on preliminary experiments, the number of estimators was set to 31 and the maximum tree depth to 38. These hyperparameters were selected to balance model complexity with generalisation ability, considering the limited dataset size. To further address overfitting and evaluate robustness, 5-fold cross-validation was applied across the dataset. These settings achieved consistent classification performance while maintaining interpretability with respect to colour space features.

### 3.2. Evaluation Index of the Performance of the Classification Models

#### 3.2.1. Definition of Evaluation Index

To evaluate the performance of classification models, we used *Precision*, *Recall*, and *F1 score*. Precision is an index that indicates the proportion of the model’s predictions that are actually correct, and is defined by the following formula:

(1) $$\mathrm{Precision}=\frac{\mathrm{TP}}{\mathrm{TP}+\mathrm{FP}}$$

High precision means less false positives.

Recall is an index that indicates the proportion of actual positive data that are correctly predicted as positive, and is defined by the following formula:

(2) $$\mathrm{Recall}=\frac{\mathrm{TP}}{\mathrm{TP}+\mathrm{FN}}$$

High recall means less false negatives.

Precision and recall are generally in a trade-off relationship, and increasing one tends to decrease the other. Therefore, the F1 score is the harmonic mean of the two and is an index for evaluating balanced performance, and is defined as follows:

(3) $$F1=2\times \frac{\mathrm{Precision}\times \mathrm{Recall}}{\mathrm{Precision}+\mathrm{Recall}}$$

#### 3.2.2. Receiver Operating Characteristic (ROC) and Area Under the Curve (AUC)

The Receiver Operating Characteristic (ROC) curve is an important method for evaluating the performance of classification models. It visualises the relationship between the True Positive Rate (TPR) and the False Positive Rate (FPR) at different thresholds, as defined by the following equations:

(4) $$\mathrm{TPR}=\frac{\mathrm{TP}}{\mathrm{TP}+\mathrm{FN}},\;\mathrm{FPR}=\frac{\mathrm{FP}}{\mathrm{FP}+\mathrm{TN}}\;,$$

where TP represents True Positives, FN represents False Negatives, FP represents False Positives, and TN represents True Negatives. Additionally, the Area Under the Curve (AUC) represents the area under the ROC curve and is a numerical measure to evaluate the performance of the classification model as described in (5). Generally, the AUC value ranges from 0.5 to 1.0, and the higher the value, the better the classification performance. A value closer to 1 indicates an ideal classification. However, when class imbalance is significant, FPR might be underestimated, so the precision–recall curve is sometimes used instead.

(5) $$\left\{\begin{array}{cc} \mathrm{AUC}=1.0 & \;\mathrm{Ideal}\;\mathrm{classification}\;\mathrm{model}\\ 0.9\leq \mathrm{AUC}<1.0 & \;\mathrm{Very}\;\mathrm{good}\;\mathrm{performance}\;\mathrm{and}\;\mathrm{practical}\;\mathrm{classification}\;\mathrm{model}\\ 0.8\leq \mathrm{AUC}<0.9 & \;\mathrm{Good}\;\mathrm{classification}\;\mathrm{model}\\ 0.7\leq \mathrm{AUC}<0.8 & \;\mathrm{Acceptable}\;\mathrm{classification}\;\mathrm{model}\\ 0.5<\mathrm{AUC}<0.7 & \;\mathrm{Low}\;\mathrm{performance}\;\mathrm{classification}\;\mathrm{model}\\ \mathrm{AUC}=0.5 & \;\mathrm{Random}\;\mathrm{classification}\;\mathrm{model}\\ \mathrm{AUC}<0.5 & \;\mathrm{Classification}\;\mathrm{model}\;\mathrm{with}\;\mathrm{high}\;\mathrm{misclassification}\;\mathrm{rate} \end{array}\right.$$

## 4. Results

**Figure 7:**
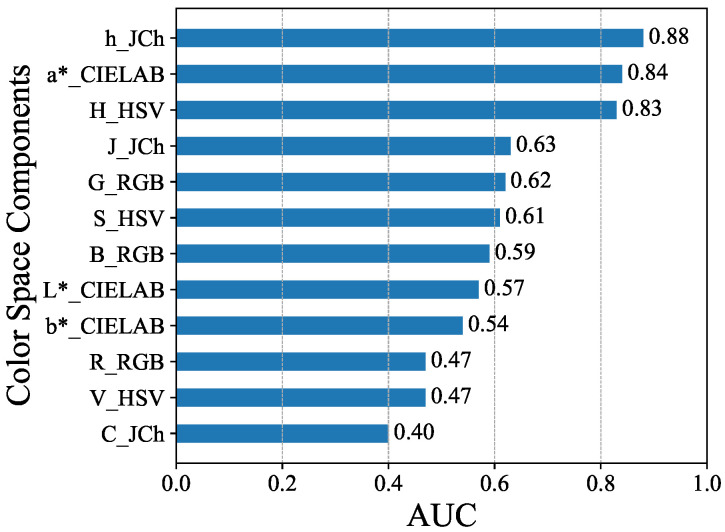
Area Under the Curve (AUC) for each component.

Figure 7 shows the results of each component and AUC. In order from top to bottom, the top three components, h_JCh = 0.88, a*_CIELAB = 0.84, and H_HSV = 0.83, showed higher performance compared to others. The performance of the other components was in the range of 0.63 to 0.40, and showed clearly lower results compared to the former.

**Figure 8:**
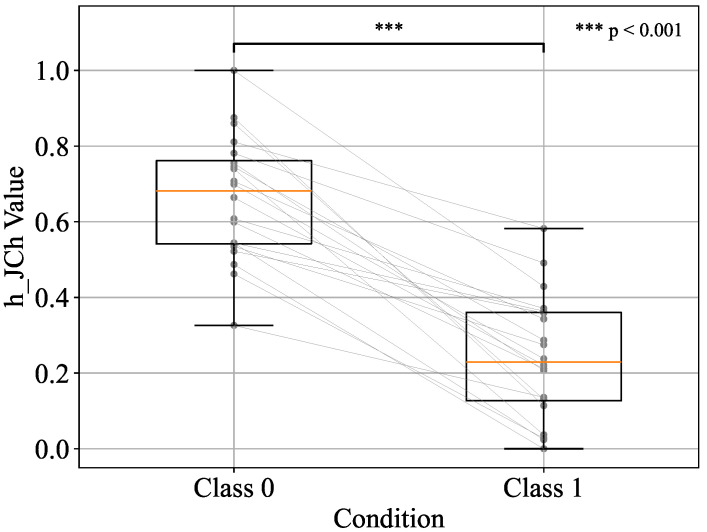
Boxplot representation of h_JCh values for Class 0 and Class 1 conditions.

**Figure 9:**
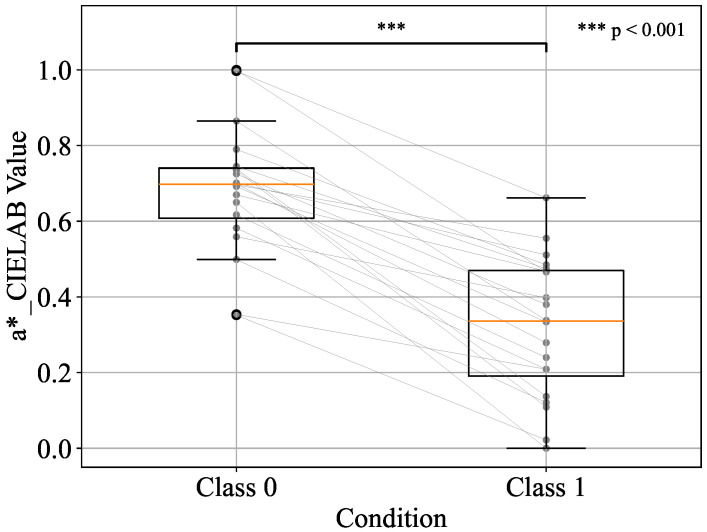
Boxplot representation of a*_CIELAB values for Class 0 and Class 1 conditions.

**Figure 10:**
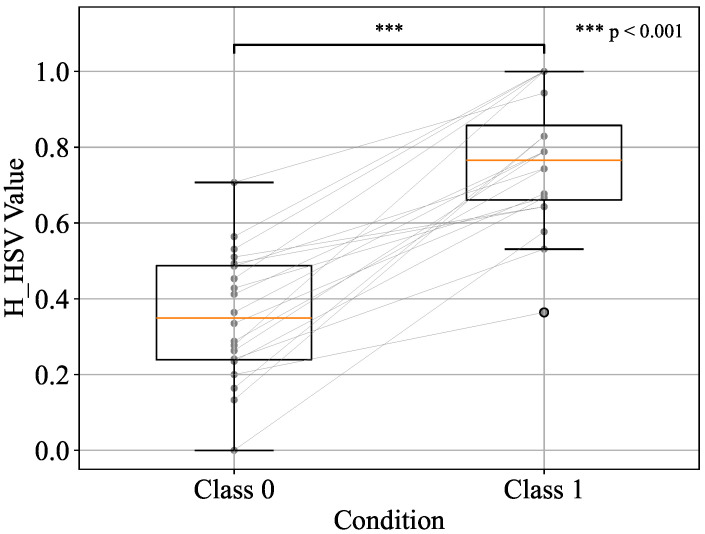
Boxplot representation of H_HSV values for Class 0 and Class 1 conditions.

Before performing the paired t-test, the normality of Class 0 and Class 1 was assessed using the Shapiro–Wilk test for each of the top three components—h_JCh, a*_CIELAB, and H_HSV. The results confirmed that all components met the assumption of normality (p>0.05), thereby justifying the application of the parametric test. Based on these results, a paired two-sample t-test was then conducted on the standardised values between the two classes. The distributions of each component are visualised using box plots in Figure 8, Figure 9, and Figure 10, respectively.

In all cases, statistically significant differences were observed in the average values of the two classes. Specifically, the results were t=10.23,p<0.001 for h_JCh, t=10.27,p<0.001 for a*_CIELAB, and t=−10.08,p<0.001 for H_HSV. These findings indicate that the two classes have different distributions and that there are significant differences in the measurements.

In addition to statistical significance, effect sizes were also calculated using Cohen’s *d*, which quantifies the magnitude of the differences between classes. Cohen’s *d* values exceeding 0.8 were interpreted as large, with values above 2.0 indicating extremely large effects according to conventional benchmarks [31]. The results showed large effect sizes for all three components: d=2.28 for h_JCh, d=2.29 for a*_CIELAB, and d=−2.29 for H_HSV. These values indicate a strong discriminative ability and practical significance in distinguishing between before- and after-pressure application.

Furthermore, the following changes were observed for each component before and after applying pressure. h_JCh decreased after pressure application, the redness weakened, and a shift toward yellow and green was observed. Similarly, a*_CIELAB also decreased after applying pressure, and the redness weakened and a shift toward green was confirmed. On the other hand, H_HSV increased after pressure application, resulting in an emphasis on visual hue changes such as redness and blueness. Finally, machine learning was performed using these top three components as explanatory variables, and the classification performance was evaluated using the area under the receiver operating characteristic curve (AUC) and F1 score.

**Figure 11:**
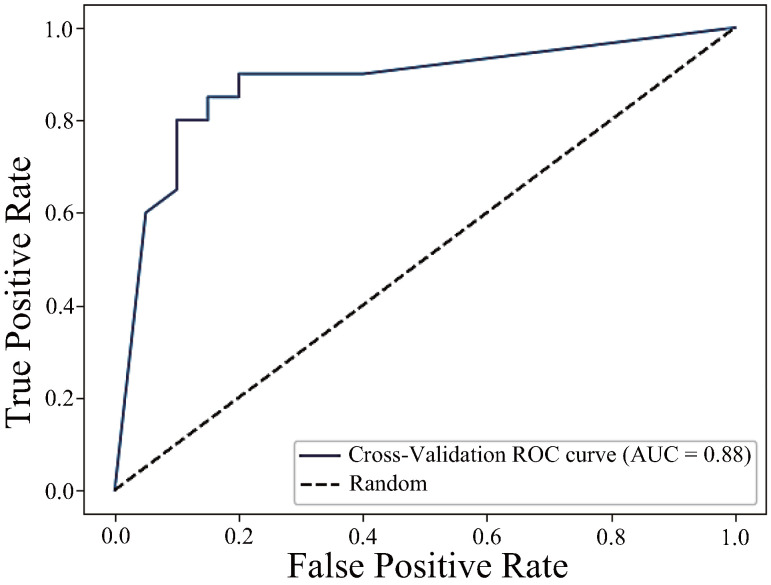
ROC curve using the h_JCh component (AUC = 0.88).

First, the ROC curve for the model using the best component, h JCh, is shown in Figure 11. This model had a good classification performance, with an AUC of 0.88, and high precision, recall, and F1 score of 0.85.

**Figure 12:**
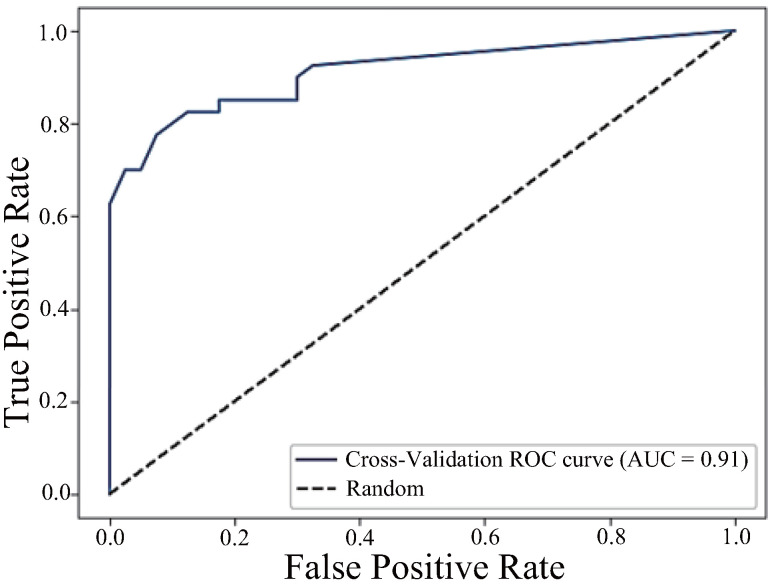
ROC curve combining the components h_JCh and a*_CIELAB (AUC = 0.91).

Next, the ROC curve for the model, with the top two components h_JCh and a*_CIELAB as explanatory variables, is shown in Figure 12. This model had an even higher AUC of 0.91, and precision, recall, and F1 score of 0.83, making it the best model overall in classification performance.

**Figure 13:**
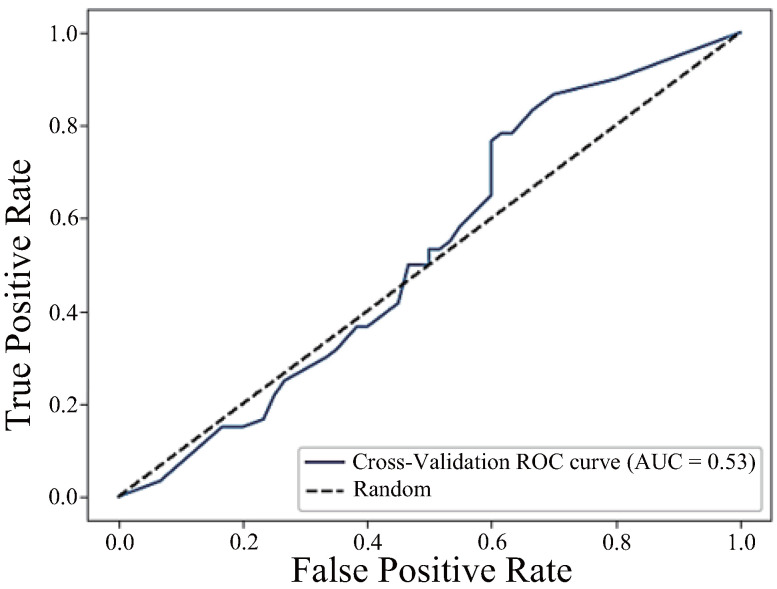
ROC curve combining the components h_JCh, a*_CIELAB, and H_HSV (AUC = 0.53).

Finally, Figure 13 shows the ROC curve for the model using the top three components h_JCh and a*_CIELAB, and H_HSV as explanatory variables. This model had a low AUC of 0.53, a precision of 0.52, a recall of 0.50, and an F1 score of 0.51, making it a model with low classification performance.

These results show that the model combining h_JCh and a*_CIELAB showed the best classification performance. The results of the three models are shown in Table 2.

## 5. Discussion

In this study, we analysed the skin colour change caused by pressure using different colour space components, and applied supervised machine learning to reveal that h_JCh, a*_CIELAB, and H_HSV were particularly effective in identifying skin colour changes before and after pressure. These specific components were important indicators for capturing changes in microcirculation caused by pressure, and showed high discrimination ability as machine learning features.

**Figure 14:**
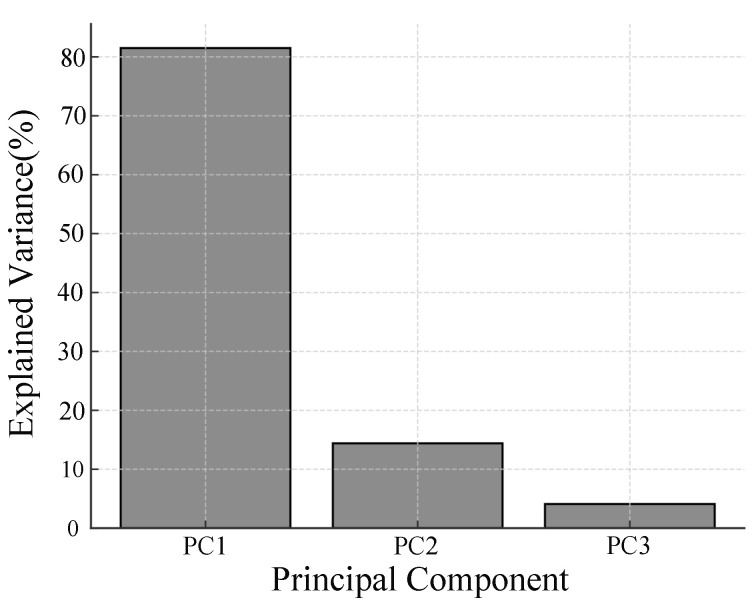
Explained variance by each principal component, showing that over 95% of the variance is explained by the first two components, supporting dimensionality reduction without significant information loss.

**Figure 15:**
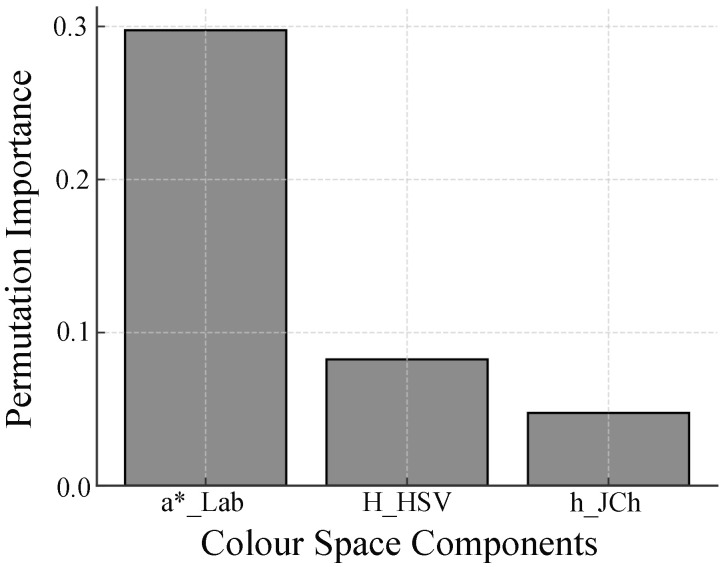
Permutation importance of each color space feature, indicating that a*_CIELAB had the highest contribution within the classifier’s predictions, followed by H_HSV and h_JCh.

To further validate our feature selection and address potential redundancy, we conducted additional analyses, including Pearson correlation, principal component analysis (PCA), and permutation importance evaluation, using the prepared dataset. To confirm the consistency and complementarity of the selected features, the Pearson correlation matrix was calculated and is presented in Table 3. The results revealed strong negative and positive correlations among the features, indicating potential redundancy. Specifically, Pearson correlation analysis revealed that a*_CIELAB had the strongest linear correlation with the labels, followed by h_JCh and H_HSV, supporting its relevance in detecting pressure-induced skin colour changes. The principal component analysis was then performed to evaluate the dimensionality, and the explained variance is shown in Figure 14, demonstrating that over 95% of the variance could be explained using two principal components, indicating that dimensionality reduction could be achieved without significant information loss. Furthermore, the feature contributions to the classifier were evaluated using permutation importance, as depicted in Figure 15, which confirmed that a*_CIELAB had the highest contribution within the classifier’s predictions, followed by H_HSV and h_JCh. Although all three features contributed to the model, the inclusion of highly correlated features, such as h_JCh and H_HSV, may lead to redundancy and impair classification performance due to multicollinearity. While the permutation importance analysis indicated that feature removal did not decrease classification performance, this result is attributable to redundancy among these features rather than indicating that they are unimportant for prediction. Collectively, these analyses support the robustness of the selected features, with a*_CIELAB demonstrating strong linear and model-agnostic contributions, while h_JCh provided effective non-linear discrimination within the classification model, consistent with its high AUC performance. This confirms that, despite redundancy due to multicollinearity, the extracted color space features remain critical for accurately capturing pressure-induced microcirculatory changes.

The reason why h_JCh showed the highest classification performance is that the JCh colour space is a perceptually uniform colour space developed based on CIECAM02 and is designed to match the characteristics of human vision [26]. Due to this characteristic, it is believed that the difference in hue is highly correlated with human perceptual differences, making it suitable for quantifying colour changes observed on the skin surface. Specifically, changes in skin colour accompanying the application and release of pressure are closely related to changes in blood flow dynamics. In the process observed in the skin of Japanese adult subjects, with predominantly Fitzpatrick skin types II–III, venous blood observed from the epidermis in a normal state appears pale blue. When blood flow is inhibited by applying pressure, the skin temporarily turns white [32]. This phenomenon occurs because the blood in the capillaries decreases, weakening the redness, and it was confirmed that h_JCh decreased after pressure. This is thought to be a reflection of the decrease in redness due to the decrease in blood flow, as a change in hue angle. Furthermore, after pressure is released, a transient congestion occurs due to the reperfusion phenomenon, and the process of changing from a slightly light yellow to red is observed [33]. The a*_CIELAB value also decreased after pressure, reflecting the weakening of the redness on the red-green axis. In addition, the H_HSV value increased after pressure, indicating a change from red to blue or yellow, reflecting a decrease in redness.

In this study, the hue change before and immediately after the pressure is released was clearly detected as an angle change in h_JCh expressed in a polar coordinate system, which is thought to have enabled the machine learning model to effectively learn nonlinear hue changes.

Next, the model combining the top two h_JCh and a*_CIELAB showed the highest classification performance (AUC = 0.91) because h_JCh captures the overall change in hue in a polar coordinate system, while a_Lab expresses the change in the red-green axis in a Cartesian coordinate system [34]. Therefore, it is considered that the feature was able to appropriately quantify the reduction in redness of the epidermis due to the change in blood flow immediately after the pressure was released. In addition, h_JCh and a*_CIELAB have different characteristics but are complementary to each other, and it is considered that the combination of these two components with different characteristics captures the change in skin colour due to the change in blood flow from multiple angles and improves the classification accuracy.

On the other hand, the significant decrease in classification performance (AUC = 0.53) in the model combining h_JCh, a*_CIELAB, and H_HSV may be due to the influence of multicollinearity. Specifically, since both h_JCh and H_HSV are components that represent hue and have similar information, they may have been treated as redundant features in the machine learning model, causing overfitting.

Our Pearson correlation analysis revealed a strong negative correlation between h_JCh and H_HSV (r = –0.982), indicating that these components capture overlapping hue-related information but in opposite directions. This high degree of informational redundancy may have interfered with the model’s learning process and reduced its generalisation performance [35].

While h_JCh and a_CIELAB also exhibited a positive correlation (r = 0.783), they likely provided complementary information across polar and Cartesian colour spaces, which may have contributed to the improved classification accuracy.

Although our current analysis focused on pairwise correlations, we recognise that more comprehensive assessments of feature redundancy, such as the Variance Inflation Factor (VIF), will be necessary in future work to guide more robust and interpretable feature selection. This will help mitigate multicollinearity and enhance model stability in diverse clinical settings, where skin appearance may be influenced by a broader range of physiological and environmental factors.

In particular, considering clinical applications, the increase in FN is a significant problem. This actually increases the risk of overlooking patients with problems in skin blood flow. To address this issue, it is important to remove redundant features and select only features that provide complementary information. Moreover, given the clinical objective of early detection and prevention of pressure ulcers, a detection strategy that prioritizes sensitivity is desirable. From this perspective, a modest increase in false positives may be acceptable if it facilitates earlier interventions, such as enhanced skin care or timely repositioning. Since pressure ulcer formation often becomes irreversible once tissue damage occurs, adopting a highly sensitive approach can support proactive clinical decisions and reduce patient risk even during the early stages of circulatory compromise. The results of this study showed that the combination of h_JCh and a*_CIELAB is optimal.

To better contextualise this result within the broader landscape of skin perfusion assessment methods, we compared our approach to established techniques. Compared to semi-automatic CRT, which requires manual compression and observer-dependent interpretation of skin colour recovery, the proposed method quantitatively captures pressure-induced skin colour changes using hue-sensitive components such as h_JCh and a*_CIELAB. These colour space features allow for consistent and objective assessment without direct contact. While IPPG also offers non-contact and continuous monitoring, it relies on photoplethysmographic signals that are sensitive to motion artifacts and ambient lighting. In contrast, our approach directly leverages robust colour representations that reflect microcirculatory changes, enabling early detection of perfusion impairment through simple imaging under controlled illumination. A summary comparison of these methods is provided in Table 4.

Future challenges include verification of subjects with different ethnic backgrounds, multi-layering of biological information by integrating with hyperspectral image analysis, and development of an automatic feature extraction method using deep learning. By addressing these challenges, it is expected that the accuracy and scope of application of this method can be further expanded. Furthermore, by achieving consistency between empirical visual assessment by nurses and quantitative data, it is expected to be applied to clinical applications such as bedsore risk assessment and skin blood flow monitoring as a new diagnostic index. In particular, the adoption of the JCh colour space is expected to have a high affinity with the medical judgment process in that it bridges human perception characteristics and numerical data.

Compared to conventional contact measurement methods, the non-contact measurement method using a camera used in this study reduces the risk of infection and is highly useful in medical settings where infection control is important. In addition, accurate measurements are possible because pressure on the measurement site does not affect skin reactions, and the device is also suitable for continuous monitoring with consideration given to patient comfort. These features are thought to contribute not only to reducing the burden on patients but also to improving the quality of medical care.

While this study primarily aimed to establish proof of concept, we acknowledge that practical considerations for real-world clinical implementation must be addressed in future work. These include cost-effectiveness, integration with existing nursing workflows, and user training requirements. Furthermore, the pathway to regulatory approval as a medical device will need to be explored. As this study was limited to a relatively small cohort of 20 healthy Japanese adults with predominantly Fitzpatrick skin types II–III, we acknowledge that this sampling does not fully reflect the diversity of real-world clinical populations. In particular, individuals with diabetes mellitus, peripheral vascular disease, or immobility-related circulatory compromise may exhibit altered baseline pigmentation and reperfusion responses. Moreover, color-based detection methods are inherently sensitive to skin tone variations, which may affect model generalizability across ethnic backgrounds. Our future studies will also have a retrospective study using photographic records and skin care assessments obtained during routine clinical care, which would allow us to evaluate skin colour changes sustained under pressure at clinically relevant anatomical locations, thereby better approximating real-world risk factors. To expand the scope of subjects, we are planning to include at risk patients, as well as a broader variety of Fitzpatrick skin types. Although the sample size was limited due to ethical restrictions, the study successfully demonstrated proof-of-concept under tightly controlled conditions. Future research with larger and more diverse populations will be essential to validate the generalizability of the findings. To achieve this, we also plan to explore the application of domain adaptation techniques, such as transfer learning or adaptive normalization, to enhance the proposed method’s robustness and to mitigate baseline tone effect across subjects from various diverse ethnic populations. The model’s generalizability will also be evaluated across multiple clinical settings in the future. To enhance translational potential, we plan to evaluate how this technology can be embedded into current pressure ulcer prevention protocols, particularly as a supplementary tool for early skin assessment and proactive care.

## 6. Conclusions

In this study, we proposed a method to binary classify subtle colour changes associated with pressure changes using colour space components. It was found that h_JCh showed the highest classification performance when using a single component, and h_JCh and a*_CIELAB showed the highest classification performance when using a combination of components. This suggests that the method is effective in preventing skin disorders caused by pressure, as it allowed for a quantitative evaluation of changes in skin colour before pressure application. In the future, we plan to continue analysis using clinical data and build a high-quality model. Lastly, further research is needed to consider differences in skin colour due to ethnic backgrounds due to the experiments conducted on people with only Japanese skin characteristics.

## Figures and Tables

**Table 1:** Abbreviations of explanatory variables.

Abbreviation	Variable	Abbreviation	Variable	Abbreviation	Variable
R	R_RGB	G	G_RGB(G)	B	B_RGB(B)
H	H_HSV	S	S_HSV	V	V_HSV
L*	L*_CIELAB	a*	a*_CIELAB	b*	b*_CIELAB
J	J_JCh	C	C_JCh	h	h_JCh

**Table 2:** Evaluation metrics for each model.

Model	AUC	Precision	Recall	F1 Score
h_JCh	0.88	0.85	0.85	0.85
h_JCh + a*_CIELAB	0.91	0.83	0.83	0.83
h_JCh + a*_CIELAB + H_HSV	0.53	0.52	0.50	0.51

**Table 3:** Pearson correlation matrix among the color space components used for classification.

	H_HSV	a*_CIELAB	h_JCh
H_HSV	1.00	−0.78	−0.98
a*_CIELAB	−0.78	1.00	0.78
h_JCh	−0.98	0.78	1.00

**Table 4:** Comparison of skin perfusion assessment methods.

Method	Invasiveness	Temporal Characteristics	Reported Classification Performance
Semi-automatic CRT [4]	Contact (manual pressure application and release)	Limited to discrete post-compression response; typically observer-triggered	Not reported
Imaging PPG (iPPG) [7]	Non-contact (camera-based photoplethysmography)	Suitable for continuous monitoring of periodic signals; less responsive to transient events	Not reported
Proposed method (h_JCh + a*_CIELAB)	Non-contact (RGB image-based)	Capable of capturing frame-by-frame colour changes immediately after pressure release	AUC = 0.91

## Data Availability

The data presented in this study are only available upon request from the authors subject to certain limitations to maintain ethics, data privacy, and anonymity for the test participants.

## Abbreviations

The following abbreviations are used in this manuscript:

ROI	Region of Interest
TPR	True Positive Rate
FPR	False Positive Rate
ROC	Receiver Operating Characteristic
AUC	Area Under the Curve
IEC	International Electrotechnical Commission
RGB	Red, Green, Blue
sRGB	Standard RGB color space
HSV	Hue, Saturation, Value
CIE	International Commission on Illumination
CIECAM02	CIE Color Appearance Model 2002
CRT	Capillary Refill Time
WHO	World Health Organization
AHA	American Heart Association
PPG	Photoplethysmography
IPPG	Imaging Photoplethysmography
NIRS	Near-infrared Spectroscopy
ScvO_2_	Central Venous Oxygen Saturation
ACM	Asymmetric Colour matching
GA	Genetic Algorithm
RF	Random Forest
VIF	Variance Inflation Factor
PCA	Principal Component Analysis
